# *Staphylococcus Aureus* Carriage in French Athletes at Risk of CA-MRSA Infection: a Prospective, Cross-sectional Study

**DOI:** 10.1186/s40798-017-0094-z

**Published:** 2017-08-16

**Authors:** E. Couvé-Deacon, D. Postil, O. Barraud, C. Duchiron, D. Chainier, A. Labrunie, N. Pestourie, P.M. Preux, B. François, M.C. Ploy

**Affiliations:** 1 University Limoges, UMR, 1092 Limoges, France; 2 INSERM, UMR, 1092 Limoges, France; 3 CHU Limoges, Laboratory of Bacteriology-Virology-Hygiene, 2 avenue Martin Luther King, 87042 Limoges, cedex France; 4 INSERM, CIC-1435, F-87000 Limoges, France; 5 CHU Limoges, Centre d’Epidémiologie de Biostatistique et de Méthodologie de la Recherche, Limoges, France

**Keywords:** *Staphylococcus aureus*, Community associated, Panton Valentine leukocidin, Nasal carriage, Throat carriage, Sports team

## Abstract

**Background:**

*Staphylococcus aureus* (SA) is a leading cause of infectious diseases in sports teams. In recent decades, community-associated SA (CA-SA) strains have emerged worldwide and have been responsible for outbreaks in sports teams. There are very few data on the prevalence of these strains in France, and none on the carriage among athletes.

**Methods:**

We conducted a cross-sectional study to determine the SA carriage proportion among athletes practicing sports at risk for CA-SA infection in a French county, and determined the methicillin-resistant and/or CA-SA proportion. We also analyzed SA carriage according to risks factors and studied the SA clonality in a sample of our population.

**Results:**

We included 300 athletes; SA carriage proportion was 61% (*n* = 183) and one was MRSA carrier (0.33%). The MRSA strain belonged to the clonal complex ST5. None of the strain produced Panton Valentine Leucocidin, and we did not find clonal distribution within the teams. Interestingly, we found a high throat-only carriage (*n* = 57), 31.1% of the SA carriers.

**Conclusion:**

We found a high SA carriage with a local epidemiology quite different than that reported in a similar population in the USA. Further studies on SA carriage should include throat sampling.

**Trial registration:**

The approved protocol was registered on ClinicalTrial.gov, NCT01148485.

## Keypoints

- The prevalence of *S. aureus* carriage in at-risk athletes was high but MRSA carriage was low and there was no community associated SA carriage.
- We found no clear evidence of clonal transmission. SA carriage out of outbreak period is probably complex, resulting of a balance between host susceptibility and endogenous flora with skin contact transmission within a sports team.
- Throat-only carriage represented a high percentage of the SA carriers which substantially inflated the nose carriage. Studies on SA carriage should include throat sampling for a better accuracy.
- Staphylococcal carriage prevention in this population could consist of simple hygiene measures promotion, known to efficiently halt SA transmission in sports teams.

## Background

*Staphylococcus aureus* (SA) is a leading cause of both community-acquired and healthcare-associated infections. Community-associated methicillin-resistant *S. aureus* (CA-MRSA) emerged worldwide during the last decade. CA-MRSA causes skin and soft-tissue infections such as multiple abscesses among healthy individuals, as well as life-threatening necrotizing pneumonia in children and young adults [1]. There is a strong epidemiological link between CA-MRSA causing deep primary skin infections and Panton-Valentine leukocidin (PVL). PVL-producing SA strains have a peculiar antibiotic susceptibility profile and are more susceptible to antimicrobials as compared to healthcare-associated MRSA [2]. The prevalence of CA-MRSA seems to be low but is increasing in Europe, although prevalence in clinical isolates vary according to the country from less than 0.5 to 15% across published studies [3].

Skin-to-skin contact and poor hygiene are risk factors for CA-MRSA outbreaks, and populations at risk of CA-MRSA infection include prisoners, military personnel, and sports teams [4]. Most studies of SA infections among athletes have been conducted in the USA, and also in the UK, Germany, and Japan [5, 6]. In the USA, CA-MRSA infections among athletes have become very frequent, the most at-risk sports being those involving physical contact, such as American football, rugby, and wrestling [5, 6]. CA-MRSA infections have also been linked to sports involving less physical contact but shared equipment, such as fencing, martial arts, cross-country running, volleyball, basketball, football, baseball, and weight-lifting [5]. Other sport-related SA infection risk factors include a high body mass index, use of equipment resulting in skin abrasion, and poor personal hygiene (sharing of personal items, failure to protect skin lesions) [6].

The anterior nasal cavities are the most common SA carriage site with the oropharynx (10–50%), but skin carriage is also frequent, especially on the hands (27–90%), perineum (22–60%), and axilla (8–19%), the intestinal tract, vagina, and skin lesions are also common carriage sites [7]. Nasal carriage has been associated in literature with a higher incidence of SA infections [7]. Twenty to 25% of healthy volunteers were reported to be permanent nasal carriers, 60% intermittent carriers and 20% permanent non-carriers [8]. In the US general population, 1.3% were nasal MRSA carriers, compared to 5.4% of athletes and individuals in daily contact with MRSA carriers [9, 10].

In France, the reported SA nasal carriage rates were about one third of the general population. However, unlike the USA, there are very few data on the prevalence of CA-MRSA in France, and none on carriage among athletes at risk of CA-MRSA infection. The aim of this study was to determine the SA carriage proportion among athletes practicing physical contact sports in a French county, and the proportion and ST types of isolates resistant to methicillin and/or producing the PVL. We also analyzed SA carriage according to the sport, hygiene habits, and medical history.

## Methods

### Study Design

We conducted a cross-sectional study of a representative sample of athletes in the French county of Limousin. We selected sports at risk of CA-MRSA infection, namely rugby, wrestling, basketball, volleyball, handball, fencing, martial arts, football, weight-lifting and baseball.

Based on the reported SA nasal carriage of 27 to 37.2% in the general population but with previous rate reported around 35% in the literature (15, 29), we determined that the required population size for an expected SA carriage proportion of 35%, a precision of 8%, and a type 1 risk of 5% was 150 subjects, calculated using Nquery Advisor 7.0 (Statistical Solutions, Saugus, MA, USA). The estimated sample size anticipated a missing data rate of 10%. We used cluster sampling—the cluster being a sports team—that included all registered teams practicing the targeted sports in Limousin. The teams were randomly selected. All subjects in a selected team were eligible for enrollment. As cluster sampling doubles the required population size, we included 300 subjects.

The study, as biomedical research, was authorized by the French competent authorities and the local ethics committee (CPP). The study was carried out exactly as described in the approved protocol (Clinicaltrials.gov, NCT01148485). The study was proposed to the randomly selected sport teams. When we obtained the team agreement, an appointment during a training session was decided. During the training session, participation was proposed to all present athletes. After information and obtainment of the signed consent of the athletes, the study sampling was done.

### Inclusion and Exclusion Criteria

The inclusion criteria were age over 18 years, registration in a Limousin sport club, and practice of at least one of the selected sports. Exclusion criteria were inability to complete the study questionnaire or if sampling was not possible. Each participant signed informed consent forms and samples were collected during regular training sessions.

The athletes completed a questionnaire on demographic characteristics, sport practice, personal hygiene, and medical history.

### Microbiological Procedures

Swab samples of the nose, throat, groin and skin lesions (defined as excoriation, erosion or crust of the skin) were transported to the microbiological laboratory at ambient temperature, seeded on *S. aureus* specific chromogenic media (chromID™ *S. aureus*, bioMerieux, Lyon, France) and incubated for 48 h at 37 °C in an aerobic atmosphere. SA was identified on the basis of colony aspect, Gram staining, and catalase, and coagulase production (Pastorex®, Fumouze, Levallois-Perret, France).

The antibiotic susceptibility profile of isolates was determined as recommended in the guidelines of the Antibiogram Committee of the French Microbiology Society (CA-SFM). Methicillin susceptibility was studied with cefoxitin disks. When this failed to categorize the isolate as susceptible or resistant, the *mecA* gene (encoding methicillin resistance) was sought by real-time PCR with specific primers, the method was adapted from a previously published protocol [11]. The PVL-encoding gene was also sought by PCR in all SA isolates, as previously described [12]. MRSA strains were characterized by Multi-Locus Sequence Typing, Enright et al. method [13]. MLST is based on the sequencing of seven housekeeping genes, defining an allelic profile corresponding to a Sequence Type (ST). ST designations were those assigned by the MLST database (available from: URL: http://www.mlst.net).

An athlete was defined as a carrier if at least one strain of *S. aureus* was isolated from at least one of his or her samples, and as an MRSA carrier if at least one SA strain resistant to methicillin was detected in at least one sample.

### ERIC PCR Strain Typing

We performed ERIC PCR for strain molecular typing [14] with the ERIC2 primer. To study clonal transmission within each team and between the different teams, we choose a sample of the strains isolated. Amplification products were separated by agarose gel electrophoresis in 2.0% agarose, and visualized by u.v. transillumination. The fingerprints obtained by ERIC–PCR were visually compared. We classified strains in major clones (clone A, clone B, clone C…) that differ by two and more bands or minor clones profiles when there was only 1 or 2 bands difference (A1, A2, A3…).

### Statistical Analysis

The study database was created with Oracle-based CLINSIGHT software (https://ecrf.clinsight.fr/nepha/), notably using the CS-DESIGNER module.

Statistical analysis used SAS V9.2 software (SAS Institute Cary, NC), and a *p* value <0.05 was considered to signify statistical significance. Quantitative variables are described as the mean ± standard deviation (SD) or the median and interquartile interval. Qualitative variables are described as numbers, percentages, and 95% confidence intervals.

Carriage proportions were compared according the sport and risk factor by using the chi^2^ test or Fisher’s exact test, depending on the theoretical number. To adjust the results, we performed a multivariate logistic regression. We selected the explanatory variables with a *p* value under 0.25. The selected variables were then computed to obtain the final model, with the variable to explain being the presence or absence of SA strain in the different samples.

## Results

### Population Characteristics

Between 11 January 2011 and 10 June 2011, we studied 300 athletes, comprising 247 men (82.3%) and 53 women (17.7%). Average age was 30.4 years (±10.1), average height 176.0 cm (±7.7), average weight 76.4 kg (±13.4), and the average body mass index 24.5 (±3.5). The athletes performed at local (12.0%, *n* = 36), regional (47.3%, *n* = 142), pre-national (6.3%, *n* = 19), or national level (10.3%, *n* = 31) and these level did not apply for 72 athletes due to their sport. Only one of the 27 studied teams was professional, representing 2.7% of the 300 athletes (Table 1).

**Table 1:** General Description of the Population

Label	Mean	Std Dev	Minimum	Maximum	Lower quartile	Median	Upper quartile
Age	30.40	10.10	18.02	86.77	22.76	27.97	35.69
Height	176.04	7.75	154.00	200.00	170.00	176.00	181.00
Weight	76.38	13.44	44.00	140.00	67.00	76.00	85.00
Body Mass Index	24.53	3.52	17.20	43.20	22.10	24.20	26.10
variable	Modality			Number of subjects		Percent	
Gender	M			247		82.33	
	F			53		17.67	
linked to healthcare system profession	NO			283		95.93	
	YES			12		4.07	
Sport competition level^a^	National			31		13.6	
	Pre-National			19		8.33	
	Region			142		62.28	
	Local			36		15.79	
Professional/amateur	Professional			8		2.67	
	Amateur			292		97.33	
Sport 27 teams	Martial art			45		15.00	
	Baseball			31		10.33	
	Basketball			19		6.33	
	Football			48		16.00	
	Weight-lifting			16		5.33	
	Handball			54		18.00	
	Wrestling			11		3.67	
	Rugby			56		18.67	
	Volleyball			20		6.67	
Championship	NO			58		19.53	
	YES			239		80.47	
Number of training session per week	Less than 1			21		7.02	
	1			66		22.07	
	2			141		47.16	
	2 and +			71		23.75	

^a^This variable does not include all the sports. Sports as martial arts, wrestling, and weight lifting are not included

### SA and MRSA Carriage

Among the 300 athletes, 61.0% (*n* = 183) carried SA (95% confidence interval (95%CI) = [51.0; 70.0]). The proportion of MRSA carriage was 0.3% (*n* = 1) (95%CI = [0.0; 1.4]) overall and 0.5% (*n* = 1) (95%CI = [0.0; 1.7]) among SA carriers (*n* = 183). The MRSA strain belonged to the clonal complex ST5 and was isolated in the nose and skin lesion swabs of a handball player. None of the SA isolates produced PVL. We noticed a high variability of team SA carriage, from 0.0% in two karate teams (*n* = 10 and *n* = 3) to 100% in a wrestling team (*n* = 11) and a baseball team (*n* = 6). A rugby team had the largest number of members (*n* = 39) and presented a SA carriage of 74.4% (*n* = 29).

Interestingly, throat carriage was more frequent (*n* = 143, 47.7%) than nasal carriage (*n* = 111, 37.0%), and throat-only carriage was frequent (*n* = 57, 19.0%) in the population. We sampled 21 skin lesions, and skin lesion-only carriage was 1.7% (*n* = 5). Multiple sampling sites increased the chances of detecting SA carriage: nasal/throat sampling identified 93.4% of carriers, while nasal/throat/groin and nasal/throat/skin lesion sampling identified 97.3% of carriers.. SA carriage was detected only in the throat, nose or groin in respectively 31.1% (*n* = 57), 12.0% (*n* = 22), and 3.2% (*n* = 8) of SA carriers (*n* = 183) (Table 2).

**Table 2:** SA Carriage According to the Sampling Sites

Location	Frequencies (% of carriers) (*n* = 183)
Nose	111 (60.6%)
Nose only	22 (12.0%)
Throat	143 (78.1%)
Throat only	57 (31.1%)
Groin	40 (21.8%)
Groin only	2 (3.2%)
Skin lesion	21 (11.5%)
Skin lesion only	5 (1.7%)
Nose and/or throat	171 (93.4%)
Nose and/or groin	121 (66.1%)
Nose and/or skin lesion	119 (65.0%)
Throat and/or groin	156 (85.2%)
Throat and/or skin lesion	151 (82.5%)
Groin and/or skin lesion	53 (29.0%)
Nose and/or throat and/or groin	178 (97.3%)
Nose and/or throat and/or skin lesion	178 (97.3%)
Nose and/or groin and/or skin lesion	127 (69.4%)
Throat and/or groin and/or skin lesion	161 (88.0%)
Nose and/or throat and/or groin and/or skin lesion	183 (100%)

### Risk Factors for SA Carriage

A history of lengthy (>4 weeks) antibiotic therapy (*p* = 0.035), use of saunas (*p* = 0.093), weight-lifting facilities (*p* = 0.152), and a history of abscess in the previous year (*p* = 0.096) had a *p* value under 0.250 (Table 3) and were included in a multivariate logistic regression model. However, after a selection step of variables none of these four variables was significantly linked to SA carriage. No significant difference in SA carriage proportion was observed between the different sports (*p* = 0.422).

**Table 3:** Risk Factors of the Athletes

A. Sport			
Factor		SA Carriers/participants (%)	*p* value (test)
Baseball		18/31 (58.1%)	*p* = 0.422 (chi^2^)
Basketball		13/19 (68.4%)	
Football		30/48 (62.5%)	
Weight-lifting		8/16 (50.0%)	
Handball		34/54 (63.0%)	
Rugby		39/56 (70.0%)	
Individual sports/martial arts (wrestling, judo, karate)		33/56 (58.9%)	
Volleyball		8/20 (40.0%)	
B. Hygiene			
Factor	Modality	SA Carriers/participants (%)	*p* value (test)
Shared equipment	NO	39/61 (63.9%)	*p* = 0.566 (chi^2^)
	YES	142/237 (59.9%)	
Shared healthcare materials	NO	149/240 (62.1%)	*p* = 0.495 (chi^2^)
	YES	32/56 (57.1%)	
Shared personal effects	NO	108/176 (61.4%)	*p* = 0.946 (chi^2^)
	YES	75/123 (61.0%)	
Skin damage during sport	Never	22/42 (52.4%)	*p* = 0.574 (Fisher)
	Rarely	57/94 (60.6%)	
	Sometimes	75/119 (63.0%)	
	Often	24/35 (68.6%)	
	Always	5/10 (50.0%)	
Sauna use	NO	176/285 (61.5%)	*p = 0.093 (chi* ^*2*^ *)*
	YES	5/13 (38.5%)	
Use of training pool	NO	180/296 (60.8%)	*p* = 1.000 (Fisher)
	YES	1/2 (50.0%)	
Weights room use	NO	154/246 (62.6%)	*p = 0.152 (chi* ^*2*^ *)*
	YES	27/52 (51.9%)	
Skin lesion disinfection	NO	77/132 (58.3%)	*p* = 0.473 (chi^2^)
	YES	103/165 (62.4%)	
Protection of skin lesions	NO	119/198 (60.1%)	*p* = 0.905 (chi^2^)
	YES	59/97 (60.8%)	
Protection of skin lesions during sport	NO	87/158 (61.4%)	*p* = 0.767 (chi^2^)
	YES	83/139 (59.7%)	
Shower/bath frequency	Every other day	23/31 (74.2%)	*p* = 0.267 (chi^2^)
	Once a day	126/211 (59.7%)	
	Several times a day	33/57 (57.9%)	
C. Medical history in the previous year			
Factor	modality	SA Carriers/participants (%)	*p* value (test)
Hospitalization	NO	156/257 (60.7%)	*p* = 0.795 (chis^2^)
	YES	27/43 (62.8%)	
Superficial skin lesions	NO	130/215 (60.5%)	*p* = 0.763 (chi^2^)
	YES	53/85 (62.4%)	
Skin abscess	NO	175/291 (60.1%)	*p = 0.096 (Fisher)*
	YES	8/9 (88.9%)	
Pneumonia	NO	181/297 (60.9%)	*p* = 1.000 (Fisher)
	YES	2/3 (66.7%)	
Antibiotic treatment	NO	158/256 (61.7%)	*p* = 0.447 (chi^2^)
	YES	21/38 (55.3%)	
Long-term antibiotics	NO	182/294 (61.9%)	*p = 0.035 (Fisher)*
	YES	1/6 (16.7%)	
Chronic disease	NO	173/281 (61.6%)	*p* = 0.440 (chi^2^)
	YES	10/19 (52.6%)	

*p* < 0.25 value are indicated in *italics*. Data is presented in three parts: A. SA carriage according to sport, B. SA carriage according to hygiene practice, C. SA carriage according to medical history in the previous year

### ERIC PCR SA Strain Typing

We selected teams to study SA clonality. We hypothesized that clone circulation within a team would be easier to detect in large team or team with the highest carriage. Fifty-nine isolates were typed. These isolates were obtained from 55 athletes in the 2 largest team rugby teams R012 (*n* = 39) and R031 (*n* = 17), with, respectively, 71.8 and 58.8% SA carriage and the two highest carriage teams, L001 (wrestling, *n* = 11) and B004 (baseball, *n* = 6) with a 100% SA carriage. We identified six major clones (clone A to clone F) and 11 minor clones profiles (Table 4). Clones A and B were found in all teams and clone F was found only in team L001. Altogether, the results did not show a clonal distribution of the SA between the teams. Furthermore, within each team, there was no evidence of a clonal transmission.

**Table 4:** Distribution of the SA clones within the Selected Teams

Team	SA Team Carriage	Clone	Number of Strains
B004	100% (6/6)		
		A	2
		B	2
		D	1
		D1	1
L001^a^	100% (11/11)		
		A	1
		A1	1
		C	5
		C1	1
		C3	1
		D	2
		F	3
R012	71.8% (28/39)		
		A	5
		A1	6
		A3	1
		A4	1
		B	1
		B1	4
		C	2
		C2	2
		C4	1
		D	2
		E	2
		E1	1
R031^a^	58.8% (10/17)		
		A	2
		A2	1
		B	3
		B1	1
		C2	3
		E	1

^a^Some athletes carried several strains; thus, we report teams with a number of studied strains higher than the number of SA carriers

B004 was a baseball team, L001 was a wrestling team and R012 and R031 were rugby teams

## Discussion

We observed an overall *S. aureus* carriage proportion of 61% (95%CI: 51.0–70.0) among 300 French athletes. A previous European study showed a carriage rate in France of 21.1% (95%CI: 17.4–25.4) among 3870 healthy patients [15], but only the nose was sampled. The prevalence of SA carriage found here in our study is higher than that observed in the general population in other studies with multiple sampling sites [16–18]. This high SA carriage among athletes is not surprising, as SA transmission is facilitated by sharing of equipment and skin-to-skin contacts. In addition, athletes are known to have poor personal hygiene during sports, as reflected by reports of CA-MRSA outbreaks in sports teams [5, 6]. We report a high variability of team SA carriage, from 0.0% in a karate team to 100% in a wrestling team and a baseball team. Clonal transmission of SA within sports teams has already been demonstrated as well as the increase of SA isolates in the sweat of SA carriers during sport practice [19]. However, the typing of the strains did not show clear evidence of clonal transmission within the studied teams. Even though skin contact is known as the main transmission risk in SA outbreaks in sports team, the SA carriage out of outbreak period is probably more complex. Host susceptibility and endogenous flora are probably in balance with skin contact transmission within a sports team.

We observed a higher carriage in the throat (*n* = 143, 47.7%) than in the nose (*n* = 111, 37.0%), as recently reported in the general population worldwide [18, 20, 21]. Interestingly, throat only carriage represented 31.1% (*n* = 57) of our carriers (*n* = 183), and represented 19% of the total population (*n* = 300). Nose positivity accounted for 37% (111/300 subjects), which is comparable with many other studies on nasal SA carriage in the general population. However, when enriched with throat-only carriers, which are often not sought for in other studies, it added 57 cases, thus inflating the prevalence to close to 60% (168/300 subjects), which is much higher than most other studies. This raises the question as to what is the genuine rate of *S. aureus* carriage in the general population if throat swabbing would also be performed and highlights the fact that *S. aureus* does not only colonize the nose [16–18]. Due to this peculiar observation we could not really compare our high carriage proportion to other studies. This implies that a combination of throat and nose sampling should be used to detect SA carriage.

In the USA, CA-MRSA is endemic. However, only one publication reported no difference in CA-MRSA nasal carriage between college athletes (1.8%) and the general population (1.5%) [22]. Based on these reports, we expected to find a CA-MRSA carriage proportion of 1.0–2.0% among French athletes. However, in our study, none carried PVL producing MRSA.

One handball player carried a PVL-negative MRSA, in the nose and skin lesion, the strain belonged to the ST5, the second more frequent clonal complex isolated in France after ST8 [23]. In athletes, MRSA carriage differed according to the studies. A study reported that 23.1% (*n* = 44/190) of high-school football players in the USA had nasal colonization with methicillin-susceptible SA, and none with MRSA [24]. On contrary, another study reported nose and skin MRSA carriage in 35.0% of 233 healthy athletes in the USA, and found that most isolates also harbored the PVL-encoding gene [16]. When our study started, most published data on MRSA carriage in the general and athlete populations came from the USA. A European study published during our study showed an MRSA carriage of 0.4% in the French general population [15], close to the 0.3% observed here in French athletes.

The link between SA carriage and outbreaks of skin and soft-tissue infections is not always clear. Outbreaks of PVL-positive MRSA infection have been reported in sports teams with high carriage of methicillin-susceptible SA (23.0 to 48.5%) but not significantly linked to CA-MRSA carriage [24–26]. In a 1-year surveillance study of MRSA nasal carriage among student athletes, colonization alone appeared insufficient to trigger outbreaks [24]. However, CA-SA infection outbreaks remain low in European athlete population compared to USA athlete population. The reported outbreaks are usually linked to poor hygiene practices and the implementation of simple hygiene measures can efficiently halt the transmission [27]. In our study, we did not find any PVL-positive MRSA carrier, nonetheless, the high SA carriage associated with skin contact during sport practice increases the risk of SA transmission between players [19] and therefore the risk of SA carriage and infection [7]. Previous study reported a higher risk of *S. aureus* carriage overtime in contact sports athletes and that efforts should focus to prevent transmission of *S. aureus* among these athletes [28]. Prevention of SA carriage and infection in that population could be achieved by simple hygiene measure promotion as skin wound disinfection and covering during practice, showering after practice and competition, using liquid soap rather than bar soap, washing clothes and equipment after practice, refraining from cosmetic body shaving, and discouraging athletes from sharing towels and personal hygiene items [29, 30]. Our data did not allow identifying risk factors for SA carriage. We found that long-term antibiotic therapy was associated with SA carriage, but this classical risk factor was no longer significant in multivariate analysis. As French data on SA carriage among athletes were lacking, we based our sample size on American data; we therefore overestimated the carriage proportion, which partly explains the lack of statistical power. Sample size was a major limitation to the study. Despite showing a high carriage, it was only out of 300 athletes. Finally, one interesting control would be to apply the same swabbing technique to a sample of the Limousin general population.

We sampled our athletes only once, mainly during the first half of the sports season. Two previous studies showed variations in MRSA carriage according to the athletic activity. In a one-year surveillance study of student football and lacrosse athletes, MRSA carriage ranged from 4.0 to 23.0%, with a peak during the period of maximal athletic activity [31]. Moreover, a 3-year surveillance study of CA-MRSA skin and soft-tissue infections in a collegiate football team, obtained evidence that variations in MRSA carriage might be related to the time of the competitive season or to outbreaks or isolated cases of CA-MRSA infection [32].

## Conclusions

In our population of French athletes, we found a high SA carriage but a low MRSA carriage. Our local epidemiology was quite different than that reported in similar types of athletes in the USA, e.g., that the USA300 clone had not spread (yet) spread among our test population. We found a very high percentage of throat-only positive cultures, which substantially inflated the nasal carriage proportion. Further studies on *S. aureus* carriage should include throat sampling.

Staphylococcal carriage prevention in that population could consist in simple hygiene measures promotion, known to efficiently halt SA transmission in sports team.

## Abbreviations

CA-MRSA: Community-associated methicillin-resistant *Staphylococcus aureus*
MLST: Multi locus sequence typing
MRSA: Methicillin-resistant *Staphylococcus aureus*
PVL: Panton Valentine leukocidin
SA: *Staphylococcus aureus*
ST: Sequence type
